# Sentinel surveillance of imported dengue via travellers to Europe 2012 to 2014: TropNet data from the DengueTools Research Initiative

**DOI:** 10.2807/1560-7917.ES.2017.22.1.30433

**Published:** 2017-01-05

**Authors:** Andreas Neumayr, Jose Muñoz, Mirjam Schunk, Emmanuel Bottieau, Jakob Cramer, Guido Calleri, Rogelio López-Vélez, Andrea Angheben, Thomas Zoller, Leo Visser, Núria Serre-Delcor, Blaise Genton, Francesco Castelli, Marjan Van Esbroeck, Alberto Matteelli, Laurence Rochat, Elena Sulleiro, Florian Kurth, Federico Gobbi, Francesca Norman, Ilaria Torta, Jan Clerinx, David Poluda, Miguel Martinez, Antonia Calvo-Cano, Maria Paz Sanchez-Seco, Annelies Wilder-Smith, Christoph Hatz, Leticia Franco, for TropNet

**Affiliations:** 1 Department of Medicine and Diagnostics, Swiss Tropical and Public Health Institute, Basel, Switzerland; 2 University of Basel, Switzerland; 3 ISGlobal, Barcelona Centre for International Health Research (CRESIB), Hospital Clínic-Universitat de Barcelona, Barcelona, Spain; 4 Division of Infectious Diseases and Tropical Medicine, Medical Centre of the Ludwig-Maximilian-University (LMU), Munich, Germany; 5 Department of Clinical Sciences, Institute of Tropical Medicine, Antwerp, Belgium; 6 Department of Internal Medicine I, Section Tropical Medicine, University Medical Center Hamburg-Eppendorf, Hamburg, Germany; Department of Clinical Research, Bernhard Nocht Institute for Tropical Medicine, Hamburg, Germany; 7 Travel Medicine Unit, Department of Infectious Diseases, Amedeo di Savoia Hospital- ASLTO2, Torino, Italy; 8 National Referral Unit for Tropical Diseases, Infectious Diseases Department, Ramón y Cajal University Hospital, IRYCIS, Madrid, Spain; 9 Centre for Tropical Diseases, Sacro Cuore - Don Calabria Hospital, Negrar, Italy; 10 Clinical Research Unit, Department of Medicine and Diagnostics, Swiss Tropical and Public Health Institute, Basel, Switzerland; 11 Department of Infectious Diseases, Leiden University Medical Centre, Leiden, The Netherlands; 12 Tropical Medicine and International Health Unit Vall d’Hebron-Drassanes. PROSICS. Hospital Vall d’Hebron. Institut Català de la Salut, Barcelona, Spain; 13 Infectious Disease Service & Department of Ambulatory Care, University Hospital, Lausanne, Switzerland; 14 University Department of Infectious and Tropical Diseases, University of Brescia and Spedali Civili General Hospital, Brescia, Italy; 15 Travel Clinic, Department of Ambulatory Care and Community Medicine, University Hospital, Lausanne, Switzerland; 16 Microbiology Department, Hospital Vall d´Hebron. PROSICS Barcelona. Universitat Autònoma de Barcelona, Barcelona, Spain; 17 Department of Infectious Diseases and Respiratory Medicine, Charité University Medical Center, Berlin, Germany; 18 National Centre for Microbiology, Instituto de Salud Carlos III, Majadahonda, Spain; 19 Institute of Public Health, University of Heidelberg, Germany; 20 Department of Global Health and Epidemiology, Umea University, Umea, Sweden; 21 Gorgas Memorial Institute, Panama, Panama; 22 http://www.tropnet.net/

**Keywords:** Dengue, Surveillance, travel, travellers, importation, Europe

## Abstract

We describe the epidemiological pattern and genetic characteristics of 242 acute dengue infections imported to Europe by returning travellers from 2012 to 2014. The overall geographical pattern of imported dengue (South-east Asia > Americas > western Pacific region > Africa) remained stable compared with 1999 to 2010. We isolated the majority of dengue virus genotypes and epidemic lineages causing outbreaks and epidemics in Asia, America and Africa during the study period. Travellers acted as sentinels for four unusual dengue outbreaks (Madeira, 2012–13; Luanda, 2013; Dar es Salaam, 2014; Tokyo, 2014). We were able to characterise dengue viruses imported from regions where currently no virological surveillance data are available. Up to 36% of travellers infected with dengue while travelling returned during the acute phase of the infection (up to 7 days after symptom onset) or became symptomatic after returning to Europe, and 58% of the patients with acute dengue infection were viraemic when seeking medical care. Epidemiological and virological data from dengue-infected international travellers can add an important layer to global surveillance efforts. A considerable number of dengue-infected travellers are viraemic after arrival back home, which poses a risk for dengue introduction and autochthonous transmission in European regions where suitable mosquito vectors are prevalent.

## Background

Over the last decades, dengue has emerged as the most important arthropod-borne viral disease globally. Currently, almost half of the world’s population lives in endemic regions, and it is estimated that ca 390 million infections occur annually, of which 96 million cases manifest clinically. In the absence of a vaccine and due to the limited efficacy of vector control strategies, dengue has seen a 30-fold increase in disease burden over the last half century, primarily in tropical and subtropical regions of South-east Asia, the Pacific region and the Americas [1]. With increasing international tourism, dengue has also emerged as an important cause of fever in travellers returning from endemic regions, and the frequency of dengue importation to non-endemic regions such as Europe continues to increase [2,3]. This trend is paralleled by the introduction, or presence and rapid expansion, of potential mosquito vectors such as *Aedes (Stegomyia) albopictus*, which is currently present in at least 15 European countries [4]. While *A. albopictus* is present in the Mediterranean region, *A. aegypti*, the primary vector of dengue in most endemic regions of the world, is found on Madeira (where *A. albopictus* is absent) and in the Black Sea region of Russia’s Southern Federal District (Sochi region) and the neighbouring Abkhazia region of Georgia (where *A. albopictus* is also absent) [5].

Therefore, dengue does not only pose a risk to the health of the individual traveller but is also a public health problem as travellers contribute to the spread of the disease [6]. The potential threat from dengue importation to non-endemic, but vector-infested, regions has been highlighted in the recent years by cases of autochthonous dengue transmission in southern Europe [7-9] and a major outbreak with more than 2,000 autochthonous dengue cases in Madeira, Portugal from 2012 to 2013 [10]. Although a great deal of effort is made to prevent the spread of dengue viruses via infected mosquitoes by implementing mosquito abatement programmes at international airports and spraying adulticides in passenger cabins of arriving aircraft, mosquitoes as agents of spread are probably overrated and viraemic travellers are a more likely source of importation of dengue viruses [6]. Therefore, when assessing the risk of introducing dengue to non-endemic regions like Europe, the key focus of surveillance is, besides evaluation of the local prevalence and distribution of potential mosquito vector species, to evaluate the extent of imported dengue via travellers. This task was covered by the European Network for Tropical Medicine and Travel Health (*TropNet*) in the past and is currently covered by the European Travel Medicine Network (*EuroTravNet*).

In addition, sentinel surveillance of travellers represents an additional important layer in the currently still fragmentary global surveillance situation. Especially travellers returning from regions where surveillance capacities are limited can uncover outbreaks that would otherwise go unnoticed. The detection of dengue fever in 10 travellers returning from Luanda, Angola to five countries on four continents in 2013 highlights this aspect [11]. Genetic characterisation of dengue virus strains collected from different geographical locations over time via returning travellers offers the opportunity to understand the global distribution and evolution of dengue sero- and genotypes and may allow us to identify and trace virus strains with epidemic potential that pose an increased risk of introduction to non-endemic regions like Europe [12].

The aims of this study were to report the phylogeny and genetic characteristics of dengue viruses imported to Europe by returning travellers and to describe the epidemiological trends of dengue infections imported to Europe by returning travellers.

## Methods

### Study objectives, patient recruitment and sample collection

The presented data were collected within the framework of the *DengueTools* research initiative (funded by the 7th Framework Programme for Research and Technological Development of the European Commission) as part of a study conducted in research area 3, ‘Risk of dengue spreading to uninfected regions’ work package 6, ‘sentinel surveillance of imported dengue in returning travellers: trends and virus evolution’ [13]. The study was conducted as a prospective observational multi-centre study by major *TropNet* centres, enrolling patients with acute dengue infections between September 2011 and December 2014. The participating *TropNet* sites were: Antwerp (Belgium), Munich (Germany), Berlin (Germany), Hamburg (Germany), Negrar (Italy), Turin (Italy), Brescia (Italy), Leiden (the Netherlands), Madrid (Spain), Barcelona (Spain), Basel (Switzerland) and Lausanne (Switzerland). The study was approved by the responsible ethics committees at all participating study sites.

Between September 2011 and December 2014, all European residents (all age groups) returning from dengue-endemic regions and presenting with an acute dengue infection (confirmed by PCR, NS1 antigen detection or positive IgM serology at the participating study sites) no later than 7 days after onset of fever, were eligible for study inclusion. The cut-off at 7 days of illness was chosen because virus isolation after this time point becomes unlikely due to declining viraemia. After signing the informed consent form, the participants completed a questionnaire on demographic data, travel history, clinical and paraclinical data and blood serum was obtained and stored at −80 °C for latter shipment on dry ice to the National Centre of Microbiology at the Instituto de Salud Carlos III (ISCIII) in Madrid, Spain, where all samples (tested positive at the participating study sites) were processed for virus sequencing and virus isolation.

### Virus isolation, sequencing and phylogenetic analysis

Sero- and genotyping was performed by RT-PCR targeting the junction between the envelope (E) and non-structural 1 (NS1) protein genes with subsequent sequencing of 400–500 bp of the E/NS1 region for each serotype as described previously [14]. The sequences were edited and analysed in Mega 6 software [15] using maximum likelihood or neighbour-joining methods. All samples were subjected to virus isolation in C6/36 *A. (S.) albopictus* mosquito cells.

## Results

### Demographic data of enrolled cases

Between September 2011 and December 2014, a total of 673 laboratory-confirmed imported dengue cases were seen at the participating study sites, of whom 244 (36%) presented during the acute phase of the infection (≤ 7 days after onset of symptoms). Of the 244 patients, 242 consented to participate in the study and were enrolled. Table 1 shows the number of cases enrolled per country and their travel profile. Some 128 (53%) of enrolled patients were male (median age: 41 years; range: 17–73 years) and 114 (47%) were female (median age: 32 years; range: 17–73 years).

**Table 1:** European imported dengue cases enrolled in the study, by country and travel profile, 2012–14 (n = 242)

	Number of cases	%
Enrolled cases by country		
Belgium	25	10.3
Germany	73	30.2
Italy	37	15.3
The Netherlands	13	5.4
Spain	74	30.6
Switzerland	20	8.3
Travel profile of cases		
Individual tourists	132	54.5
Package tourists	50	20.7
Visiting friends and relatives	29	12.0
Business travellers	23	9.5
European overseas residents/expatriates	8	3.3

### Geographical origin of imported dengue cases

**Figure 1:**
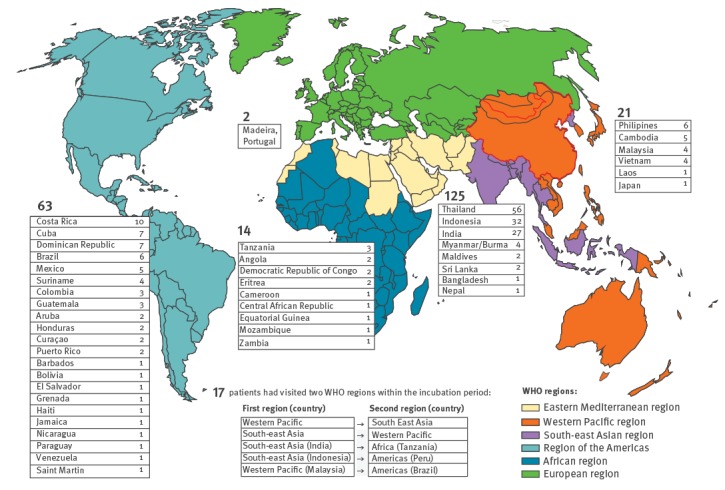
Geographical distribution of imported dengue cases, by WHO region, 2012–14 (n = 242)

Figure 1 shows the geographical background of the imported dengue cases by World Health Organization (WHO) region: 125 cases (52%) were imported from the South-east Asian region, 63 cases (26%) from the Americas, 21 cases (8%) from the Western Pacific region, 14 cases (6%) from the African region, two cases (0.8%) from Madeira (Portugal) and 17 cases (7%) had visited two different WHO regions in the incubation period. Of the 17 cases who had visited two WHO regions, 14 travellers had visited countries of the neighbouring WHO regions South-east Asia and Western Pacific and three cases had visited two non-neighbouring WHO regions in the incubation period. Virus isolation and sequencing was successful in two of the three cases who had visited two non-neighbouring WHO regions in the incubation period. 

WHO: World Health Organization.

Note: The figure shows absolute numbers and not incidence rates and thus reflects the popularity of travel destinations rather than the risk.

### Pattern of imported dengue cases during the study period

**Figure 2:**
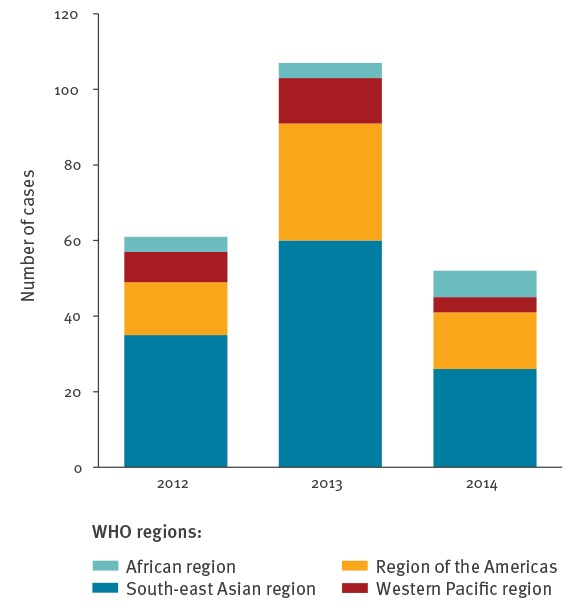
Geographical pattern of imported dengue cases, by WHO region, 2012–14 (n = 242)

Because ethical clearance was obtained by the study sites at different time points, the cases collected in 2011 (n = 21) were not homogeneously enrolled into the study and therefore excluded from the trend analysis. In 2012, 2013 and 2014, all participating study sites recruited patients. Figure 2 depicts the overall importation pattern of acute dengue fever from January 2012 to December 2014. No seasonal importation pattern was observed from any endemic region (data not shown).

WHO: World Health Organization.

Note: The figure shows absolute numbers and not incidence rates and thus reflects the popularity of travel destinations rather than the risk.

### Proportion of travellers presenting with acute/viraemic dengue infection

**Figure 3:**
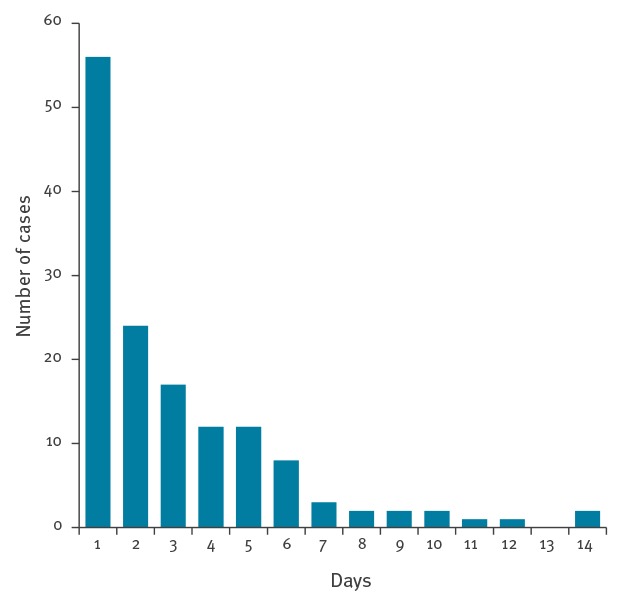
Time between onset of symptoms and returning back home in returning travellers developing dengue fever, 2012–14 (data available from 143 travellers)

To assess the overall risk of dengue importation by potentially infectious/viraemic travellers (who may introduce the virus to regions of Europe where suitable mosquito vectors are present) we assessed the proportion of acutely ill/viraemic travellers among all imported dengue cases seen at the participating study sites. Of 673 imported dengue cases seen during the study period, 244 (36%) presented during the acute phase of the infection (≤ 7 days after onset of symptoms). The remaining 64% of patients presented later than 7 days after onset of illness (mainly for follow-up or confirmation of the diagnosis) but we have no further details on these patients as our aim was to include acutely ill, potentially viraemic travellers. Among the 242 study participants presenting with acute dengue (symptoms ≤ 7 days), 87 (36%) already developed symptoms while travelling and 155 (64%) became symptomatic after returning home. Figure 3 shows the delay between onset of symptoms and returning home. Of the 242 acute dengue cases, 160 (66%) were positive by PCR and virus isolation followed by sero- and genotyping was successful in 141 (58%) cases.

### Virus isolation, sequencing and phylogenetic analysis

**Figure 4:**
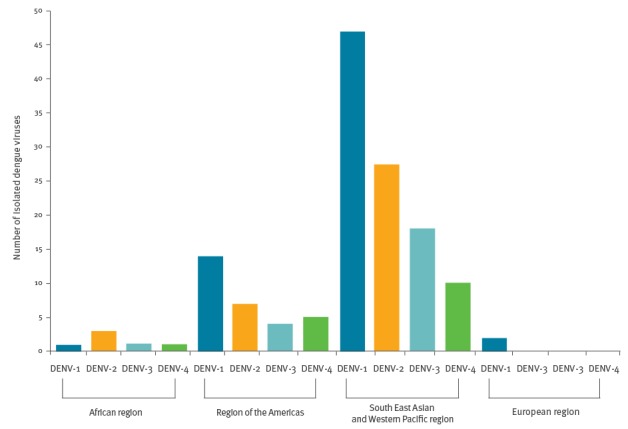
Distribution of imported dengue virus serotypes, by WHO region, 2012–14 (n = 141)

Of the 141 virus isolates that could be typed, DENV-1 was identified in 46% (n = 65), DENV-2 in 26% (n = 37), DENV-3 in 16% (n = 23) and DENV-4 in 11% (n = 16) of cases (Tables 2 and 3). All four dengue serotypes were imported by travellers, irrespective of which region they had visited (Figure 4). The two dengue infections acquired within the European region were diagnosed in travellers returning from Madeira during the local dengue outbreak in 2012–13 and were due to DENV-1 (Figure 4).

**Table 2:** Study participants with imported dengue virus infection (n = 673) and PCR-positive cases wit available virus sequence (n = 141), 2012–14

Recruitment of study participants and processing of samples	Number of cases
All dengue cases seen at the participating study sites during the study period	673
Laboratory-confirmed acute dengue cases presenting within ≤ 7 days after onset of symptoms to one of the study sites ( = enrolled patients according to inclusion criteria)	242
PCR-positive acute dengue cases	160
Acute dengue cases where sequencing and virus isolation was successful	141

**Table 3:** Virological results, returning travellers with dengue virus infection, 2012–14 (n = 141)

**Serotypes (n = 141)**	**n**	**%**	**Genotypes**
DENV 1	65	46	I (Asian) and V (America-Africa)
DENV 2	37	26	America-Africa, Cosmopolitan and Asian I
DENV 3	23	16	I, II, III and IV
DENV 4	16	11	I and II

#### Dengue serotype 1 (DENV-1)

Overall, DENV-1 (n = 65) was the serotype most frequently detected in the study population. All isolated DENV-1 strains belonged to two of the five genotypes previously described [16-18]: genotype I (Asian) and genotype V (America-Africa). The most notable findings were:

*Genotype I (Asian):* All genotype I (Asian) virus strains (n = 42) were isolated from travellers returning from Asia. One virus was isolated from a traveller returning from Japan in September 2014; the case was linked to a local outbreak with ca. 160 reported autochthonous cases affecting Tokyo from August to September 2014 [19].

*Genotype V (America-Africa):* As reported previously [14], genotype V (America-Africa) strains (n = 23) show a vast geographical distribution and we isolated virus strains from travellers returning from the Americas, Africa and Asia. Three cases were notable: two virus strains from travellers returning from Madeira during the dengue epidemic in 2012 and 2013 and one virus strain from a traveller returning from Angola during the dengue outbreak in 2013 [20].

#### Dengue serotype 2 (DENV-2)

The isolated DENV-2 strains (n = 37) grouped into three different genotypes that currently are of high epidemiological interest: genotypes America-Asia (n = 9), Cosmopolitan (n = 23) and Asian I (n = 5).

*Genotype America-Asia*: All strains were isolated from travellers returning from the Americas.

*Genotype Cosmopolitan:* We isolated two virus strains from travellers returning from Tanzania in 2014 at the time of an ongoing dengue outbreak in Dar es Salaam and neighbouring regions (personal communication: Boillat N, Sep 2015). The isolated virus strains clustered in a lineage different from the strains introduced to Africa in the early 1980s.

#### Dengue serotype 3 (DENV-3)

Four different genotypes of DENV-3 (n = 23) were detected during the study period: genotypes I (n = 7), II (n = 5), III (n = 8) and IV (n = 23), suggesting a broad expansion and diversity of circulating DENV-3 strains. The most notable findings were the isolation of two genotype III strains from travellers returning from Cuba in 2013 and 2014, suggesting epidemic circulation of these strains, and the isolation of a genotype III strain from a traveller returning from Burkina Faso in 2013, a region for which data on dengue endemicity are not available.

#### Dengue serotype 4 (DENV-4)

DENV-4 strains were the least frequently isolated virus strains in our study. All DENV-4 strains imported from the Americas (n = 16) belonged to genotype II, the main genotype circulating in the region since its introduction in 1982 [14]. All detected strains of DENV-4 from Asia (n = 16) were genotypes I (n = 7) and II (n = 9). Most notably, we isolated a genotype II strain from a traveller returning from Angola which showed 98% homology to strains currently circulating in Brazil, confirming previous data suggesting that the 2013 DENV-4 outbreak in Luanda was caused by a virus strain introduced from Brazil [21].

## Discussion

The observed geographical pattern of the origin of imported dengue by international travellers was in line with previous reports from *TropNet* (1999–2001: South-east Asian/Western Pacific region: 53.4%, American region 36.5%, African region: 10.3%) [22] and data from the *GeoSentinel* network (2000–10: South-east Asian/western Pacific region: 67%, American region: 28%; African region: 5%) [23].

The peak of imported dengue cases observed in 2013, compared with 2012 and 2014, was mainly attributable to the increase in cases imported from the Americas and South-east Asia and is in line with the isochronal epidemiological trend observed in these regions: The Pan American Health Organisation (PAHO) reported 1,120,902 cases in 2012, 2,386,836 cases in 2013 and 1,176,529 cases in 2014 which occurred in the American region [24]. Although neither the WHO figures for the South-east Asian region nor the official figures from Thailand (which accounts for the majority of dengue cases imported from the South-east Asian region to Europe) were traceable, accessible online media sources reported that in 2013, Thailand experienced its worst dengue epidemic in more than two decades [25], followed by a significant decline in cases in 2014 [26]. From 2012 to 2014, the overall geographical pattern of the origin of imported dengue to Europe remained unchanged and the importation pattern over the years appears to match the epidemiological situation in endemic regions. However, it should be kept in mind that the absolute numbers of dengue infections in travellers returning from different destinations primarily reflect the popularity of travel destinations and cannot provide incidence rates or an assessment of infection risk, as data on the exact number of travellers to the different regions (denominator) are not available.

Among the imported dengue cases, three travellers (1.2%) had visited two non-neighbouring WHO regions during the possible incubation period, highlighting the potential role of international travellers in transcontinental spread of DENV strains. This is corroborated by the DENV strains isolated from two of these three travellers: The virus isolated from a patient who travelled from Indonesia to Peru points to Indonesia as the most likely place of acquisition and the identified genotype has not yet been known to circulate in Peru. The virus isolate from the patient who travelled from Malaysia to Brazil points to Brazil as the most likely place of acquisition. (Note: the detailed phylogenetic analysis of all DENV isolated within the framework of this study is envisaged but currently pending). 

When looking at the number of dengue cases enrolled into the study over time, no seasonal pattern of importation was detected. However, the number of enrolled cases may have been insufficient to see a seasonal trend. The European Centre for Disease Prevention and Control (ECDC) reports a seasonal trend of imported dengue cases in Europe, increasing during the summer and autumn months (June–October) and peaking in August [27]. This may be explained by the European summer holiday season with the corresponding increase of international travel during this time of the year as well as the epidemiological peak of dengue cases at the major holiday destination South-east Asia from June to September [28]. Mathematical modelling of the likelihood of dengue importation to Europe (taking into account dengue seasonality in the countries from which dengue could be imported, the number of reported dengue cases imported into Europe and the volume of airline travellers arriving from dengue-affected areas internationally) concluded that the risk of dengue importation is greatest in August, September and October [29]. Entomological monitoring in the Mediterranean region indicated that the development period for *A. albopictus* starts in April and closes in October/November with activity peaks from June/July to September [30,31]. The peak activity of *A. albopictus* populations in the south of Europe thus coincides with the seasonal peak of imported dengue cases in Europe which increases the risk of autochthonous transmission [29,32]. Case reports of autochthonously acquired dengue in Croatia and the south of France in August and September in the past years support this prediction [7-9]. We found that more than a third of travellers who are infected with dengue in endemic regions either return to Europe during the acute phase of the infection or become symptomatic after returning back home. More than half of the patients presenting with acute dengue infection were viraemic when seeking medical care. If we equate viraemia with risk of transmission, we can conclude that at least 58% (141/242) of dengue patients presenting during the acute phase of infection may pose a potential risk to initiate autochthonous transmission in vector-infested regions of Europe. Travellers returning to regions where no *A. albopictus* is prevalent will, even if viraemic, not pose any relevant risk for authochtonous transmission. The used cut-off of 7 days for study enrolment was a pragmatic decision and does not exclude that some patients may be viraemic beyond that period. Thus, the observed proportion of viraemic cases should be seen as a minimum. Of note, almost half of the included 242 cases were enrolled by *TropNet* sites in Spain (Barcelona) and Italy (Brescia, Torino and Verona) where *A. albopictus* is prevalent.

Worldwide surveillance of circulating DENV strains is crucial for the understanding of transmission patterns and for tracking the emergence and spread of virus strains (especially those with high epidemic potential) around the world. However, currently global surveillance data remain fragmentary. This is especially true for resource-poor regions where no or only limited local surveillance data are available. Sentinel surveillance of international travellers returning with DENV infections from such regions has been suggested as a valuable tool for filling these current data gaps [6,14] and the phylogenetic analysis of our isolated DENV strains confirms this: We detected all four DENV serotypes in travellers returning from Asia, the Americas and Africa and identified the main genotypes and epidemic lineages causing outbreaks and epidemics during the study period (the DENV-1 genotypes America-Africa and Asian, the DENV-2 genotypes Cosmopolitan and Asian I and the DENV-3 genotype III). We also picked up changes in DENV strain circulation, e.g. we isolated a genotype I Asia strain in a traveller returning from Indonesia in 2013 at the same time as local reports describing a shift from the predominantly circulating cosmopolitan genotype strains to Asian genotype strains [33].

For regions where currently only scarce or no regional surveillance data are available, virus characterisation revealed several interesting findings: 

Firstly, although 1,430 (clinical) dengue cases were officially reported in Cuba in 2013 and 2,522 in 2014, no dengue serotypes have been reported to PAHO/WHO for those years [24]. In our study, more than 10 years later, we detected circulation of dengue serotype 3 (genotype III) in Cuba in 2013 and 2014. According to PAHO, other Hispanic Caribbean countries did not report this serotype in those years. The last detection of dengue 3 (genotype III) in Cuba was during a big epidemic in 2001 and 2002 [14,34].

Secondly, we identified dengue virus circulating in countries in Africa that have so far rarely reported dengue. We isolated a genotype III DENV-3 strain from a traveller returning from Togo and Burkina Faso, a region where, besides one recent case report of a genotype III DENV-3 infection in a returning German traveller [35], circulation of dengue had been unknown. We also identified a dengue virus strain imported during the dengue outbreak in Angola in 2013. In a previous report, the outbreak was thought to be due to an endemic virus strain that had been circulating in West Africa for many years [20]. However, our analysis points towards an importation of a dengue virus from Brazil, consistent with a report by researchers from Portugal [21]. Furthermore, we isolated DENV from travellers returning from Tanzania during the dengue outbreak affecting Dar es Salaam and neighbouring regions in 2014 [36]. The isolated virus strains clustered in a lineage different from the strains introduced in Africa in the early 1980s, suggesting recent introduction from Asia.

Thirdly, our sentinel surveillance picked up a dengue outbreak in Europe: the 2012/13 outbreak in Madeira, Portugal [10].

Finally, the genotype I (Asian) DENV-1 isolate we isolated from a traveller returning from Japan in September 2014 was linked to a local outbreak with ca. 160 reported autochthonous cases affecting Tokyo in August and September 2014 [19]. The only previous autochthonous transmission of dengue in Japan was reported in 2013, when a DENV-2 strain was isolated in a returning German traveller [37], although no concurrent local cases were reported at that time and autochthonous transmission of dengue had not been reported in Japan for 70 years [19]. Despite Japan's temperate climate, increasing travel between Japan and dengue-endemic areas, combined with more suitable climate and environmental drivers for dengue transmission, have made such an outbreak possible [38]. 

In conclusion, our data demonstrate that epidemiological and virological data obtained from dengue-infected international travellers can add an important layer to global dengue surveillance efforts.
